# Optimizing Hexose Utilization Pathways of *Cupriavidus necator* for Improving Growth and L-Alanine Production under Heterotrophic and Autotrophic Conditions

**DOI:** 10.3390/ijms25010548

**Published:** 2023-12-31

**Authors:** Lei Wang, Huiying Luo, Bin Yao, Junhu Yao, Jie Zhang

**Affiliations:** 1 College of Animal Science and Technology, Northwest A&F University, Xianyang 712100, China; wangleiwray@126.com (L.W.); binyao@caas.cn (B.Y.); 2 State Key Laboratory of Animal Nutrition and Feeding, Institute of Animal Science, Chinese Academy of Agricultural Sciences, Beijing 100193, China; luohuiying@caas.cn

**Keywords:** alanine, *Cupriavidus necator*, cell density, hexose utilization pathway, metabolic engineering, poly-β-hydroxybutyrate

## Abstract

*Cupriavidus necator* is a versatile microbial chassis to produce high-value products. Blocking the poly-β-hydroxybutyrate synthesis pathway (encoded by the *phaC1AB1* operon) can effectively enhance the production of *C. necator*, but usually decreases cell density in the stationary phase. To address this problem, we modified the hexose utilization pathways of *C. necator* in this study by implementing strategies such as blocking the Entner–Doudoroff pathway, completing the phosphopentose pathway by expressing the *gnd* gene (encoding 6-phosphogluconate dehydrogenase), and completing the Embden–Meyerhof–Parnas pathway by expressing the *pfkA* gene (encoding 6-phosphofructokinase). During heterotrophic fermentation, the OD_600_ of the *phaC1AB1*-knockout strain increased by 44.8% with *pfkA* gene expression alone, and by 93.1% with *gnd* and *pfkA* genes expressing simultaneously. During autotrophic fermentation, *gnd* and *pfkA* genes raised the OD_600_ of *phaC1AB1*-knockout strains by 19.4% and 12.0%, respectively. To explore the effect of the *pfkA* gene on the production of *C. necator*, an alanine-producing *C. necator* was constructed by expressing the NADPH-dependent L-alanine dehydrogenase, alanine exporter, and knocking out the *phaC1AB1* operon. The alanine-producing strain had maximum alanine titer and yield of 784 mg/L and 11.0%, respectively. And these values were significantly improved to 998 mg/L and 13.4% by expressing the *pfkA* gene. The results indicate that completing the Embden–Meyerhof–Parnas pathway by expressing the *pfkA* gene is an effective method to improve the growth and production of *C. necator*.

## 1. Introduction

*Cupriavidus necator* H16 (formerly known as *Ralstonia eutropha* H16) is a chemolithoautotrophic Gram-negative bacterium that serves as a model organism for the natural synthesis of poly-β-hydroxybutyrate (PHB). It has been shown to accumulate up to 90% of PHB based on dry weight under restricted nutrition [1]. Its broad carbon source utilization spectrum, detailed genomic information, and efficient gene editing system [2,3] position *C. necator* as a promising candidate for applications in environmental protection and bio-resource conversion [4]. Studies have shown that *C. necator* can utilize waste oil, lignocellulose-derived sugar, and inedible rice to produce PHB [5,6,7]. Additionally, several kinds of biofuels, like alcohol [8] and fatty acids [9], have also been successfully synthesized in *C. necator* by metabolic engineering. With H_2_ providing reducing power and O_2_ acting as the electron acceptor, *C. necator* fixed CO_2_ through the Calvin–Benson–Basham cycle and flowed it into the central carbon metabolic pathway [10]. Recent studies have modified *C. necator* to convert CO_2_ into high-value products like glucose, acetoin, and 2-hydroxyisobutyrate [11,12,13]. These findings demonstrate that *C. necator* is a versatile microbial chassis for high-value biochemical production from CO_2_.

**Figure 1:**
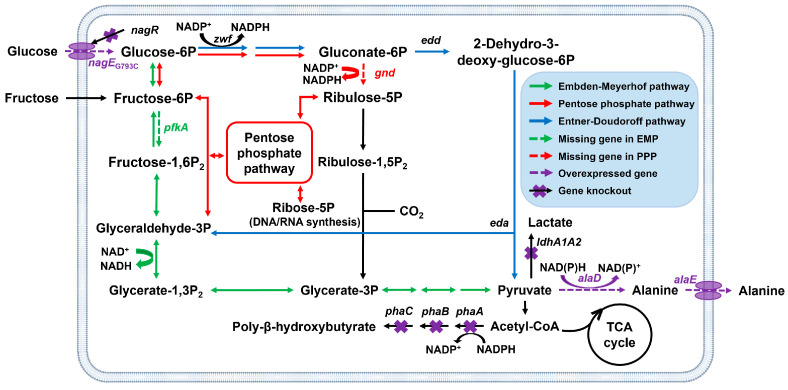
Hexose utilization pathways and metabolic engineering of *C. necator* for alanine production. The endogenous ED pathway was blocked by the knockout of glucose-6-phosphate 1-dehydrogenase (encoded by the *zwf* gene). The EMP pathway and PP pathway were completed by expressing the *pfkA* gene (encoding 6-phosphofructokinase) and *gnd* gene (encoding 6-phosphogluconate dehydrogenase) from *E. coli*, respectively. The *phaC1AB1* operon was knocked out to increase the supply of precursors. The *alaD* gene (encoding L-alanine dehydrogenase) and *alaE* gene (encoding an alanine exporter in *E. coli*) were heterologously expressed for the synthesis and export of L-alanine. *nagE*, encoding a subunit of a putative GlcNAc-specific phosphotransferase system; *nagR*, encoding a putative GntR-type transcriptional regulator of *nagE*; *edd*, encoding phosphogluconate dehydratase; *eda*, encoding 2-keto-3-deoxy-6-phosphogluconate aldolase; *ldhA1A2*, encoding D-lactate dehydrogenase; *phaA*, encoding acetyl-CoA acetyltransferase; *phaB*, encoding acetoacetyl-CoA reductase; *phaC*, encoding poly(3-hydroxybutyrate) polymerase.

In nature, the Embden–Meyerhof–Parnas (EMP) pathway and the pentose phosphate (PP) pathway are the main central carbon metabolism systems for most microbes. These pathways convert glucose into pyruvate while providing essential energy and precursors to maintain the basic metabolisms, such as DNA replication and protein synthesis. Generally, the EMP pathway collaborates with the PP pathway to regulate energy balance and coenzyme supply [14]. However, the 6-phosphofructokinase (encoded by the *pfkA* gene) in the EMP pathway and 6-phosphogluconate dehydrogenase (encoded by the *gnd* gene) in the PP pathway are absent in *C. necator* [15], indicating that hexose is mainly metabolized through the Entner–Doudoroff (ED) pathway during heterotrophic fermentation (Figure 1). Compared with the EMP pathway, the ED pathway offers a shorter and faster metabolic process and provides additional NADPH, although it has a lower adenosine triphosphate (ATP) supply. The glyceraldehyde 3-phosphate generated in the ED pathway not only follows the downstream of the EMP pathway but also participates in the PP pathway (Figure 1) to generate ribose 5-phosphate and erythrose 4-phosphate, which serve as precursors for nucleic acid and phospholipid syntheses, respectively. Consequently, it is hypothesized that the incomplete EMP and PP pathways in *C. necator* may inhibit cell growth during fermentation.

Under the regulation of the *phaC1AB1* operon (encoding three genes catalyzing the generation of PHB from acetyl-CoA), *C. necator* consumes acetyl-CoA as precursor and NADPH as coenzyme to form PHB, which accumulates in cells as the main energy storage material [16,17]. During metabolic engineering, knocking out the *phaC1AB1* operon is common practice to divert sufficient carbon flux towards product synthesis. However, this approach often leads to a significant reduction in cell density, since the knockout of the *phaC1AB1* operon may cause metabolic disorders of carbon flux, energy supply, and coenzyme balance [18]. Noting that all these factors are involved in different glycolytic pathways, this study first explored the effect of regulating hexose utilization pathways on the growth of PHB-knockout *C. necator*.

L-alanine is an aliphatic amino acid with an annual global demand of approximately 50,000 tons [19]. L-alanine is the only amino acid with a sweet taste and can be used as a sweetener, freshener, and seasoning agent in food [20,21]. As a glucogenic amino acid, L-alanine plays an important role in the treatment of diabetes and related research [22]. The catalytic synthesis of L-alanine from pyruvate by alanine dehydrogenase (encoded by the *alaD* gene) is a good choice for industrial production. As a model organism for PHB synthesis, *C. necator* has the ability to generate a large number of pyruvates, which provides sufficient substrates for L-alanine synthesis. The synthesis of L-alanine is directly influenced by pyruvate supply and coenzyme balance (Figure 1). Therefore, an alanine-producing *C. necator* was constructed in this study to validate the effect of hexose utilization pathways on alanine production. The purpose of this study was to develop a regulating strategy for *C. necator* in enhancing the production of high-value products based on pyruvate, relieving growth inhibition caused by *phaC1AB1* operon knockout, and laying the foundation for *C. necator* low-carbon industrial production through autotrophic fermentation.

## 2. Results

### 2.1. Effect of Blocking the ED and PHB Synthesis Pathways on Growth

To avoid potential interference with foreign gene expression, the H16_*A0006* gene, encoding the R subunit of type I restriction enzyme, was first knocked out in the wild-type *C. necator* to obtain strain CnΔ6. Strain CnΔ6ΔPHB was easily obtained by knocking out the *phaC1AB1* operon of strain CnΔ6. To block the ED pathway (Figure 1), attempts were made to delete or replace the *eda* and *edd* genes (encoding 2-keto-3-deoxy-6-phosphogluconate aldolase and phosphogluconate dehydratase, respectively) with the *gnd* or *pfkA* genes, but all attempts failed. Instead, we selected the strain CnΔ6Δzwf (knocking out the *zwf1*, *zwf2,* and *zwf3* genes in CnΔ6, encoding glucose-6-phosphate 1-dehydrogenase) as the ED-pathway-blocked strain and successfully knocked out the PHB synthesis pathway, obtaining the strain CnΔ6ΔzwfΔPHB.

**Figure 2:**
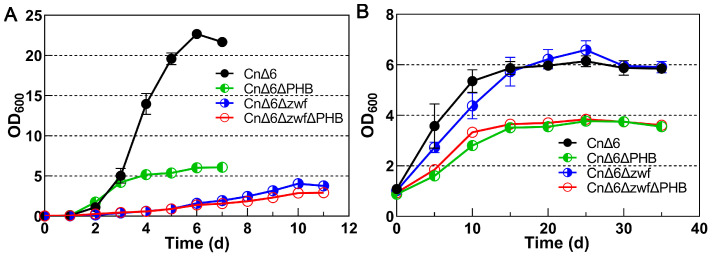
Effect of the ED and PHB synthesis pathways on the growth of *C. necator*. All of the *zwf1*, *zwf2,* and *zwf3* genes were knocked out in both CnΔ6Δzwf and CnΔ6ΔzwfΔPHB. The *phaC1AB1* operon was knocked out in both CnΔ6ΔPHB and CnΔ6ΔzwfΔPHB. (**A**) The growth curves of *C. necator* during heterotrophic fermentation with 30 g/L fructose as the sole carbon source. (**B**) The growth curves of *C. necator* during autotrophic fermentation with CO_2_ as the sole carbon source.

In the heterotrophic fermentation supplied with 30 g/L fructose as the sole carbon source, the growth rate of *C. necator* decreased notably after the knockout of *zwf* genes, and the duration of the logarithmic phase was extended from 6 to 10 days. Meanwhile, the cell density visibly decreased from 22.7 to 4.1 (Figure 2A). In contrast, *zwf* genes had no substantial impact on the growth or cell density of *C. necator* in autotrophic fermentation (Figure 2B), probably because the *zwf* genes were not involved in the carbon flux during autotrophic fermentation. In the stationary phase, the knockout of the *phaC1AB1* operon obviously reduced the cell density of CnΔ6 both in heterotrophic (from 22.7 to 6.0) and autotrophic (from 6.1 to 3.8) fermentation. In addition, no interaction was observed between the *zwf* genes and *phaC1AB1* operon affecting the growth of *C. necator* in either heterotrophic or autotrophic fermentation.

### 2.2. Effect of Completing the EMP and PP Pathways on Growth

The EMP and PP pathways are incomplete in *C. necator* for the lack of 6-phosphofructokinase and 6-phosphogluconate dehydrogenase, respectively. Here, we successfully constructed pBBR1-series plasmids (Table 1) that overexpressed the *gnd* and *pfkA* genes (both cloned from *Escherichia coli*) and electrically transformed them into ED and PHB synthesis pathway-blocking strains. Since the empty plasmid pBBR1-MCS2 had no effect on the growth of *C. necator* (Figure S1), all the differences in growth were attributed to the changes in hexose utilization pathways.

**Figure 3:**
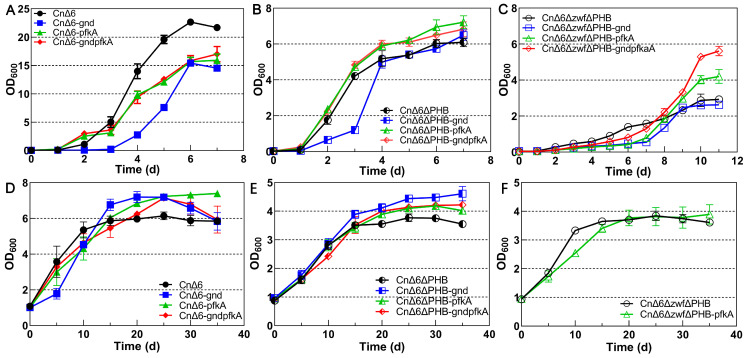
Effect of the *pfkA* and *gnd* genes on the growth of different *C. necator* strains. Both the *pfkA* and *gnd* genes were constitutively expressed with promoter P*_phaC1_* in plasmids. Heterotrophic fermentation using 30 g/L fructose as the sole carbon source and autotrophic fermentation using CO_2_ as the sole carbon source. Both the *pfkA* and *gnd* genes were cloned from *E. coli*. (**A**) The changes in the growth of CnΔ6 during heterotrophic fermentation; (**B**) the changes in the growth of CnΔ6ΔPHB during heterotrophic fermentation; (**C**) the changes in the growth of CnΔ6ΔzwfΔPHB during heterotrophic fermentation; (**D**) the changes in the growth of CnΔ6 during autotrophic fermentation; (**E**) the changes in the growth of CnΔ6ΔPHB during autotrophic fermentation; (**F**) the changes in the growth of CnΔ6ΔzwfΔPHB during autotrophic fermentation.

In the heterotrophic fermentation supplemented with 30 g/L fructose, the lag phases of CnΔ6, CnΔ6ΔPHB, and CnΔ6ΔzwfΔPHB were prolonged remarkably by expressing the *gnd* gene (Figure 3A–C). The *pfkA* gene conspicuously increased the cell densities of CnΔ6ΔPHB (from 6.1 to 7.2 in Figure 3B) and CnΔ6ΔzwfΔPHB (from 2.9 to 4.2 in Figure 3C). Overexpression of both the *gnd* and *pfkA* genes inhibited the growth of CnΔ6 (from 22.7 to 17.0 in Figure 3A). Conversely, the cell density in the stationary phase was apparently increased from 6.1 to 6.8 in CnΔ6ΔPHB-gndpfkA (Figure 3B) and from 2.9 to 5.6 in CnΔ6ΔzwfΔPHB-gndpfkA (Figure 3C). While in autotrophic fermentation, expression of the *gnd* gene no longer caused growth delay. Both the *gnd* and *pfkA* genes markedly increased the highest cell density of CnΔ6 by 18.0% and 21.3%, respectively (Figure 3D). This effect persisted in CnΔ6ΔPHB with increases of 19.4% and 12.0%, respectively (Figure 3E). After knocking out the *zwf* genes, Gnd lost its biofunction due to the lack of substrate. Therefore, only the *pfkA* gene was overexpressed to complete the EMP pathway in strain CnΔ6ΔzwfΔPHB, but no noteworthy effect on strain growth was observed (Figure 3F).

### 2.3. Construction of the Alanine-Producing Strain

Compared with fructose, glucose is cheaper and more suitable for industrial-scale production. Previous studies have reported that glucose can be utilized by *C. necator* through two approaches: expressing the exogenous *glf* gene, encoding an energy-independent glucose-facilitated diffusion transporter [23], or modifying the native N-acetylglucosamine transport system by mutation of NagE(G265R) and deletion of the *nagR* gene [24]. After construction and verification (Figure S2), the second strategy showed better growth and was applied in strain CnΔ6 for the production of alanine, resulting in strain CnΔ6RE. The *alaE* gene (encoding an alanine exporter in *E. coli*) was fused with the P*_phaC1_* promoter and inserted into the *ldhA1A2* locus of CnΔ6RE, obtaining strain CnΔ6REalaEΔldhA12, thereby promoting the export of alanine as well as blocking the synthesis of by-product lactate. Subsequently, the *phaC1AB1* operon in the genome of CnΔ6REalaEΔldhA12 was knocked out to obtain the strain CnAla.

**Figure 4:**
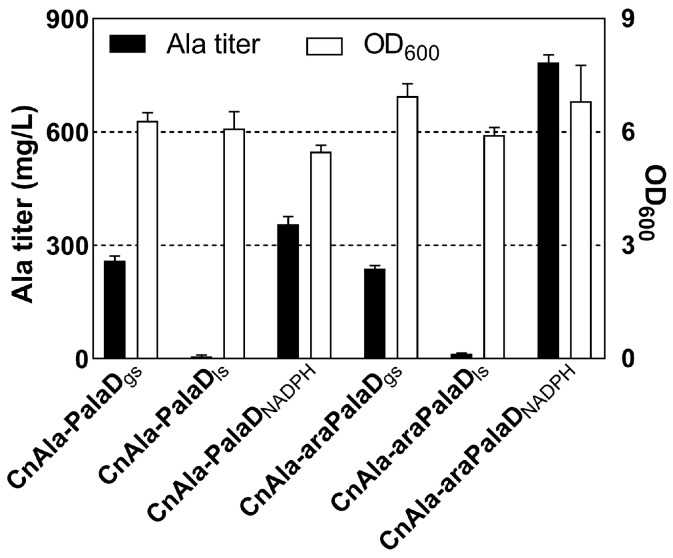
Effect of different *alaD* genes on the growth and alanine titer of strain CnAla. The highest OD_600_ and alanine titer of the strain were observed with the constitutive (by P*_phaC1_*, marked with P) or inducible (by *araC*P_BAD_, marked with araP) expression of different *alaD* genes in plasmids during heterotrophic fermentation with 30 g/L glucose as the sole carbon source. The *alaD*_gs_, *alaD*_ls_, and *alaD*_NADPH_ genes were codon-optimized from *G. stearothermophilus*, *L. sphaericus*, and *B. subtilis*, respectively.

Using NADH as a coenzyme, L-alanine dehydrogenase (encoded by the *alaD* gene) from *G. stearothermophilus* [25] and *L. sphaericus* [26] has been reported for alanine production. Scientists have engineered the *alaD* gene from *B. subtilis* to change its coenzyme preference to NADPH [27]. The above three *alaD* genes were codon-optimized and expressed in plasmids with different promoters for alanine synthesis in this study. During heterotrophic fermentation supplied with 30 g/L glucose, no alanine was detected in the strain CnΔ6REalaEΔldhA12, whether expressing any *alaD* gene with P*_phaC1_* or *araC*P_BAD_ promoter. Following the knockout of the *phaC1AB1* operon, alanine was significantly accumulated in strain CnAla (Figure 4). The constitutive and inducible expression of the *alaD*_gs_ gene in strain CnAla exhibited similar alanine production at approximately 249 mg/L. However, the *alaD*_ls_ gene was not suitable for alanine synthesis in *C. necator*. Compared with 356 mg/L of alanine titer in constitutive expression, the inducible expression of the *alaD*_NADPH_ gene in strain CnAla-araPalaD_NADPH_ (CnAlaD) showed the highest alanine titer at 784 mg/L. Furthermore, autotrophic fermentation of alanine-producing strains was also conducted in CnMM using CO_2_ as the sole carbon source, but no alanine accumulation was observed. This could be attributed to the absence of an organic carbon source in CnMM, leading to the consumption of synthesized alanine before it could be transported into the culture medium.

### 2.4. Effect of Completing the EMP and PP Pathways on Alanine Production

**Figure 5:**
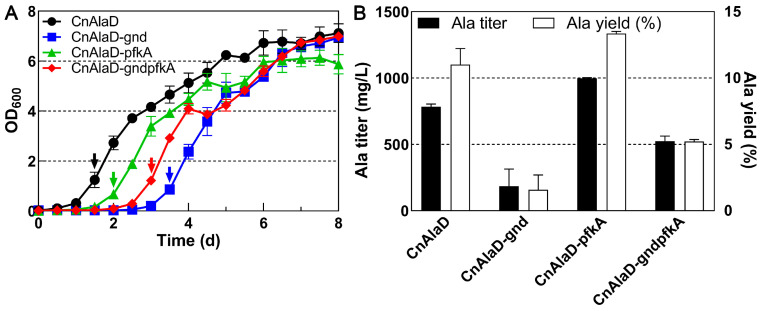
Effect of the expression of the *gnd* and *pfkA* genes on the growth and the alanine production of strain CnAlaD. Both the *gnd* and *pfkA* genes were expressed in plasmids. Heterotrophic fermentation using 30 g/L glucose as the sole carbon source. Arrows represent the time point to add inducer. (**A**) The growth curves of strain CnAlaD with different plasmids. (**B**) The highest titer and yield of glucose converted to alanine of strain CnAlaD with different plasmids.

To complete the EMP and PP pathways, the *gnd* and *pfkA* genes were separately expressed in strain CnAlaD downstream of the *alaD* gene in plasmids, along with the P*_phaC1_* promoter. Consistent with previous results, the expression of the *gnd* gene prolonged the lag period of strain CnAlaD from 1 d to 3 d without changing the highest cell density (Figure 5A). In addition, expressing the *gnd* gene also caused a significant decrease in alanine titer (from 784 mg/L to 186 mg/L, Figure 5B) and yield (from 11.0% to 1.6%, Figure 5B). In contrast, the expression of the *pfkA* gene markedly enhanced the maximum titer of alanine from 784 mg/L to 998 mg/L, and the yield of glucose converted to alanine increased from 11.0% to 13.4% (Figure 5B). The extended lag phase and decreased alanine production caused by the *gnd* gene were partially mitigated when it was co-expressed with the *pfkA* gene.

## 3. Discussion

In line with previous reports [9,16], the present study found that knocking out the *phaC1AB1* operon reduced the cell density of *C. necator* both in heterotrophic and autotrophic fermentation. This result contrasts with the findings of Lu et al. [28], who reported minimal impact on the growth in heterotrophic fermentation with the deletion of the *phaC1AB1* operon. Wang et al. [29] observed a decrease in cell density in the PHB-knockout strain using glucose as the sole carbon source, but this effect disappeared when glycerol replaced glucose. These results suggest that the carbon source might be a factor that changes the effect of the *phaC1AB1* operon knockout on the cell density of *C. necator*. Gluconate, fructose, and *N*-acetylglucosamine are the only three carbohydrates utilized by unmodified *C. necator*. Additionally, several different types of carbon sources, such as acetate, citrate, and glycerol, for instance, can also be utilized by *C. necator* [30]. Under unbalanced culture conditions, like limitation of oxygen, phosphorus, or nitrogen, a large amount of PHB was accumulated in cells of *C. necator* as energy storage material [1]. The mineral media for *C. necator* were optimized in this study, especially increasing the nitrogen concentration and the proportion of trace elements, which accelerated the growth rate of the CnΔ6 strain and significantly shortened the logarithmic phase of autotrophic fermentation from 64 d to 15 d [11]. These findings imply that various carbon sources and C/N ratios in the medium may also affect the growth of PHB-knockout strains and this merits further investigation in future studies.

The expression of the *gnd* gene partly directed the carbon flux towards the PP pathway, accompanied by the generation of a large amount of NADPH. The blocking of the PHB synthesis pathway further reduced NADPH consumption. The excess NADPH, functioning as a reductant, might induce oxidative stress in cells [31] and further manifest as growth delay or lower production property. Based on KEGG annotations [32], the *cbb_c_* operon in the chromosome and the *cbb_p_* operon in megaplasmid pHG1 were expressed independently, both capable of achieving CO_2_ fixation using NADPH as a coenzyme [33,34]. The balanced NADPH may explain why the extended lag phase caused by *gnd* gene expression only occurs in heterotrophic fermentation. Similarly, the expression of the NADPH-dependent *alaD* gene had higher alanine production, as it alleviated the stress associated with coenzyme imbalance. Apart from the imbalanced coenzyme, it has been reported that the overexpression of the *gnd* gene also reduced the intracellular acetyl-CoA concentration of *C. necator* [35], resulting in insufficient energy supply and reflected as lower alanine production in strain CnAlaD-gnd. In consequence, completing the PP pathway by expressing the *gnd* gene is not conducive to the biosynthetic modifications of *C. necator*.

In the present study, the *pfkA* gene significantly improved the cell density, alanine titer, and yield of *C. necator*. These results suggested that completing the EMP pathway by expressing the *pfkA* gene is able to boost glucose utilization efficiency in *C. necator*. The expression of the *pfkA* gene enabled the bidirectional flow of carbon flux, which originally flowed unidirectionally into the PP pathway, thereby achieving dynamic regulation of intracellular ribose (used for DNA synthesis) and pyruvate (supplied energy and carbon skeleton). In addition, the *E. coli pfkA* was found to realize the fructose oxidative decomposition through the EMP instead of the ED pathway [36]. The ATP throughput of the EMP pathway is higher than that of the ED pathway. Accordingly, it is essential to consider the ATP balance when strengthening the EMP pathway by overexpressing the *pfkA* gene. Cell growth and *L*-serine accumulation were improved after *pfkA* was over-expressed in *Corynebacterium glutamicum* [37]. The expression of *pfkA* in *Pseudomonas putida* inhibited cell growth, decreased ATP and NADPH in cells, and increased sensitivity to oxidative stress [38]. Conversely, the ATP and butanol production in *Clostridium acetobutylicum* were enhanced by overexpressing *pfkA* [39]. Hence, the appropriate *pfkA* gene expression level is crucial to demonstrate the advantage of ATP supply in the EMP pathway, ultimately resulting in faster cell growth and increased production.

The core strategy was to increase alanine production by improving the carbon flow of the alanine synthesis pathway. In terms of precursor supply, strengthening the EMP and ED pathways has great potential to increase the hexose utilization ability and enhance the supply of pyruvate [40]. Following the same rule, the expression of the *pfkA* gene increased the efficiency of pyruvate supply, thereby increasing the production of alanine in *C. necator*. In addition to precursor supply, screening for alanine dehydrogenase suitable for the chassis microbe can also redirect more carbon flow toward alanine synthesis [41]. As shown in the results of this study, NADPH-dependent alanine dehydrogenase derived from *B. subtilis* exhibited the highest alanine synthesis ability in *C. necator* when expressed with the strong arabinose-induced promoter.

In conclusion, an alanine-producing strain CnAlaD was constructed for the first time in the present study, demonstrating that *C. necator* is a valuable chassis organism for amino acid biosynthesis. Moreover, completing the EMP pathway by *pfkA* gene expression prominently improved cell density, alanine titer, and yield, which proves that the regulation of hexose utilization pathways is a powerful method to increase the production of *C. necator*, wherein the ATP balance and coenzyme balance need further exploration in future studies.

## 4. Materials and Methods

### 4.1. Strains and Culture Medium

All the strains involved in this study are shown in Table 2. The *Escherichia coli* Trans1-T1 (Transgen, Beijing, China) and S17-1 were adopted for plasmid construction and conjugation, respectively, cultivated in Luria–Bertani (LB) medium at 37 °C. Another 10 g/L fructose, as the optimal carbon source, was added to LB for the cultivation of *C. necator* at 30 °C. When solid culture was required, an additional 2% of agar was supplied to the medium. The mineral medium for *C. necator* (CnMM) was optimized from a previous study [42], containing 7 g/L Na_2_HPO_4_, 3 g/L KH_2_PO_4_, 5 g/L (NH_4_)_2_SO_4_, 0.3 g/L NaHCO_3_, 0.2 g/L MgSO_4_·7H_2_O, 0.001 g/L CaSO_4_, and 3 mL/L trace element. The trace element was redesigned according to previous studies [43,44,45], including 5 g/L ferric citrate, 1 g/L CaCl_2_, 0.6 g/L NiSO_4_·7H_2_O, 0.5 g/L ZnSO_4_·7H_2_O, 0.3 g/L CoCl_2_·6H_2_O, 0.3 g/L H_3_BO_3_, 0.22 g/L MnSO_4_·H_2_O, 0.2 g/L Na_2_MoO_4_·2H_2_O, and 0.2 g/L CuSO_4_·5H_2_O. A sole carbon source, like fructose, glucose, or CO_2_, was added into CnMM to support the growth of strains. Kanamycin (50 μg/mL for *E. coli*, 200 μg/mL for *C. necator*) and gentamicin (10 μg/mL for *C. necator*) were added to the medium if necessary.

### 4.2. Plasmid Construction

All plasmids and primers designed for this study are listed in Table 1 and Table S1, respectively. The target gene fragments and promoters were obtained by PCR using primers with homologous arms, overlapped to the linearized pBBR1 plasmid (by *EcoRI*), and assembled using the Gibson assembly. For the genomic editing of *C. necator* by homologous recombination, the upstream and downstream homologous arms (about 600 bp) of the editing site were amplified and assembled to the linearized pK18mobsacB plasmid (by *EcoRI* and *SmaI*). All constructed plasmids were verified by sequencing before enrichment and electroporation.

### 4.3. Strain Modification

Transconjugation [8] was adopted in the plasmid transformation of *C. neacator*, where the recombinant plasmid of pK18mobsacB was electro-transformed to *E. coli* S17-1 first and then integrated into the genome of *C. necator* after 24 h of co-culture. The single-cross-over colony of *C. necator* was screened using kanamycin and verified by PCR. A positive colony was then counter-selected in an LB solid plate with 50 g/L of sucrose [11]. The double-crossover colony was identified by colony PCR and sequencing.

In order to produce alanine and complement the EMP and PP pathways, plasmids carrying *alaD*, *pfkA,* or *gnd* genes were transformed into electrocompetent cells from *C. necator* by electroporation in a 2 mm cuvette with 2300 V of voltage, 25 μF of capacitance, and 200 Ω of resistance. Transformed strains were spread on LB solid medium supplemented with kanamycin after 2 h cultivation in a 30 °C shaker at 200 r/min with 1 mL of LB, and then the colonies that appeared on the plate were verified by colony PCR.

### 4.4. Heterotrophic Fermentation

*C. necator* was heterotrophically cultivated in 100 mL shake flasks containing 50 mL of CnMM, supplying 30 g/L of fructose or glucose as the sole carbon source. Strains were enriched overnight in LB, centrifuged, washed with phosphate-buffered saline (pH = 7.4), and then inoculated into flasks with an initial OD_600_ of 0.03. The heterotrophic fermentation was carried out in a shaker at 30 °C and 200 r/min. Cell density and alanine accumulation were measured every 24 h with 3 replicates per strain.

### 4.5. Autotrophic Fermentation

Autotrophic fermentation was performed in 250 mL anaerobic bottles, each containing 50 mL of CnMM. The residual gas in the anaerobic bottle was sufficiently replaced every 24 h with a fresh gas mixture (H_2_:CO_2_:O_2_ = 8:1:1 in volume) at 1 L/min for 3 min, using the mixed gas charging system (Figure S3) to ensure the carbon and energy supply [29]. The inoculation process was the same with heterotrophic fermentation, except for the initial OD_600_ at 1.0. Autotrophic fermentation was also carried out in a 200 r/min shaker at 30 °C. Samples were taken every 5 d to determine cell density and alanine concentration with 3 replicates per strain.

### 4.6. Analytical Methods

For the detection of cell density, 200 μL of diluted bacterial solution was divided into 96-well plates and OD_600_ was determined with a microplate reader (BioTek, Burlington, VT, USA). The composition of the fermentation broth was detected by HPLC. Glucose was eluted with 5 mmol/L of H_2_SO_4_ at 0.5 mL/min at 35 °C using an Animex HPX-87H (9 μm, 7.8 × 300 mm) column (Bio-Rad, Hercules, CA, USA) and quantified using a refractive index detector. Alanine was analyzed using the AJS-01 Amino Acid Analysis Method Package (Shimadzu, Sakyoku, Kyoto, Japan). Specifically, alanine was first derived with o-phthalaldehyde online and then flowed into an AJS-02 (3 μm, 4.6 × 150 mm) column; subsequent gradient elution was performed using solvent A (4.5 g/L Na_2_HPO_4_·12H_2_O and 4.25 g/L Na_2_B_4_O_7_·10H_2_O, pH adjusted to 8.2 with HCl) and solvent B (water, methanol, and acetonitrile mixed at a volume ratio of 10:45:45) at a flow rate of 2 mL/min under a temperature of 50 °C; the compounds were finally identified using a UV–vis detector at 338 nm. The growth curve and alanine accumulation were summarized and analyzed with GraphPad Prism (v8.0.2, GraphPad Software, Boston, MA, USA), adopting a paired T-test or one-way analysis of variance to compare the significance of differences in two or more groups of data.

## Figures and Tables

**Table 1:** Plasmids used in this study.

Plasmids	Characteristics	Sources
pK18mobsacB	Plasmid for conjugation and genomic editing, Km^r^	Laboratory
pK18-*phaC1AB1*	pK18mobsacB-derived, for *phaC1AB1* operon deletion	This study
pK18-*nagR*	pK18mobsacB-derived, for *nagR* deletion	This study
pK18-*nagE*_G793C_	pK18mobsacB-derived, for mutation of *nagE*(G793C)	This study
pK18-*alaE*-*ldhA1A2*	pK18mobsacB-derived, for the replacement of *ldhA1A2* with *alaE* from *E. coli* fused with P*_phaC1_*	This study
pBBR1-MCS2	Wide host plasmid for gene expression, with Cpa fdx terminator at the end of MCS, Km^r^	Laboratory
*gnd*-pBBR1	*gnd* gene from *E. coli* fused with P*_phaC1_* and assembled into pBBR1-MCS2	This study
*pfkA*-pBBR1	*pfkA* gene from *E. coli* fused with P*_phaC1_* and assembled into pBBR1-MCS2	This study
*gnd*-*pfkA*-pBBR1	*pfkA* gene from *E. coli* fused with P*_phaC1_* and assembled after the *gnd* gene of *gnd*-pBBR1	This study
*alaD*_gs_-pBBR1	Codon-optimized *alaD* gene from *Geobacillus stearothermophilus* fused with P*_phaC1_* and assembled into pBBR1-MCS2	This study
*alaD*_ls_-pBBR1	Codon-optimized *alaD* gene from *Lysinibacillus sphaericus* fused with P*_phaC1_* and assembled into pBBR1-MCS2	This study
*alaD*_NADPH_-pBBR1	Codon-optimized *alaD* gene from *Bacillus subtilis*, mutated to NADPH preference, fused with P*_phaC1_*, and assembled into pBBR1-MCS2	This study
araP*alaD*_gs_-pBBR1	Codon-optimized *alaD* gene from *G. stearothermophilus* fused with *araC*P_BAD_ and assembled into pBBR1-MCS2	This study
araP*alaD*_ls_-pBBR1	Codon-optimized *alaD* gene from *L. sphaericus* fused with *araC*P_BAD_ and assembled into pBBR1-MCS2	This study
araP*alaD*_NADPH_-pBBR1	Codon-optimized *alaD* gene from *B. subtilis*, mutated to NADPH preference, fused with *araC*P_BAD_, and assembled into pBBR1-MCS2	This study
*alaD*-*gnd*-pBBR1	*gnd* gene from *E. coli* fused with P*_phaC1_* and assembled after the *alaD* gene of araP*alaD*_NADPH_-pBBR1	This study
*alaD*-*pfkA*-pBBR1	*pfkA* gene from *E. coli* fused with P*_phaC1_* and assembled after the *alaD* gene of araP*alaD*_NADPH_-pBBR1	This study
*alaD*-*gnd*-*pfkA*-pBBR1	*pfkA* gene from *E. coli* fused with P*_phaC1_* and assembled after the *gnd* gene of *alaD*-*gnd*-pBBR1	This study

**Table 2:** Bacterial strains used in this study.

Strain	Characteristics	Sources
*E. coli*		
Trans1-T1	F^−^φ80(*lacZ*)ΔM15Δ*lac*X74*hsd*R(r_k_^−^,m_k_^+^)Δ*rec*A1398*end*A1*ton*A	Transgen, Beijing, China
S17-1	*thi pro hsdR recA*; chromosomal RP4; *Tra*^+^; *Tmp*^r^ Str/Spc^r^	ATCC47055
*C. necator*		
H16	Wild-type, Gen^r^	DSM 428
CnΔ6	H16ΔH16_*A0006*	Laboratory
CnΔ6-gnd	CnΔ6 harboring plasmid *gnd*-pBBR1	This study
CnΔ6-pfkA	CnΔ6 harboring plasmid *pfkA*-pBBR1	This study
CnΔ6-gndpfkA	CnΔ6 harboring plasmid *gnd*-*pfkA*-pBBR1	This study
CnΔ6Δzwf	CnΔ6 derived, Δ*zwf1*, Δ*zwf2*, Δ*zwf3*	Laboratory
CnΔ6ΔPHB	CnΔ6 derived, Δ*phaC1AB1*	This study
CnΔ6ΔPHB-gnd	CnΔ6ΔPHB harboring plasmid *gnd*-pBBR1	This study
CnΔ6ΔPHB-pfkA	CnΔ6ΔPHB harboring plasmid *pfkA*-pBBR1	This study
CnΔ6ΔPHB-gndpfkA	CnΔ6ΔPHB harboring plasmid *gnd*-*pfkA*-pBBR1	This study
CnΔ6ΔzwfΔPHB	CnΔ6Δzwf-derived, Δ*phaC1AB1*	This study
CnΔ6ΔzwfΔPHB-gnd	CnΔ6ΔzwfΔPHB harboring plasmid *gnd*-pBBR1	This study
CnΔ6ΔzwfΔPHB-pfkA	CnΔ6ΔzwfΔPHB harboring plasmid *pfkA*-pBBR1	This study
CnΔ6ΔzwfΔPHB-gndpfkA	CnΔ6ΔzwfΔPHB harboring plasmid *gnd*-*pfkA*-pBBR1	This study
CnΔ6RE	CnΔ6 derived, Δ*nagR nagE*_G793C_, able to use glucose	This study
CnΔ6REalaEΔldhA12	CnΔ6RE-derived, *ldhA1A2*::P*_phaC1_*-*alaE*	This study
CnAla	CnΔ6REalaEΔldhA12-derived, Δ*phaC1AB1*	This study
CnAla-PalaD_gs_	CnAla harboring plasmid *alaD*_gs_-pBBR1	This study
CnAla-PalaD_ls_	CnAla harboring plasmid *alaD*_ls_-pBBR1	This study
CnAla-PalaD_NADPH_	CnAla harboring plasmid *alaD*_NADPH_-pBBR1	This study
CnAla-araPalaD_gs_	CnAla harboring plasmid araP*alaD*_gs_-pBBR1	This study
CnAla-araPalaD_ls_	CnAla harboring plasmid araP*alaD*_ls_-pBBR1	This study
CnAla-araPalaD_NADPH_ (CnAlaD)	CnAla harboring plasmid araP*alaD*_NADPH_-pBBR1	This study
CnAlaD-gnd	CnAla harboring plasmid *alaD*-*gnd*-pBBR1	This study
CnAlaD-pfkA	CnAla harboring plasmid *alaD*-*pfkA*-pBBR1	This study
CnAlaD-gndpfkA	CnAla harboring plasmid *alaD*-*gnd*-*pfkA*-pBBR1	This study

## Data Availability

The original data are included in the paper and can be obtained from the corresponding author upon reasonable request.

## Supplementary Materials

The following supporting information can be downloaded at: https://www.mdpi.com/article/10.3390/ijms25010548/s1.
