# Supplementary material for: Optimizing Hexose Utilization Pathways of Cupriavidus necator for Improving Growth and L-Alanine Production under Heterotrophic and Autotrophic Conditions
Source: Int J Mol Sci. 2023 Dec 31;25(1):548. doi: 10.3390/ijms25010548 (PMC10778655; doi:10.3390/ijms25010548)
Supplement: Supplementary file 1 [file ijms-25-00548-s001.zip › ijms-2785081-supplementary.pdf]

Supplementary materials

Optimizing hexose utilization pathways of *Cupriavidus necator* for improving growth and L-alanine production under heterotrophic and autotrophic conditions

Lei Wang [1,2], Huiying Luo [2], Bin Yao [1,2], Junhu Yao [1,*] and Jie Zhang [2,*]

College of Animal Science and Technology, Northwest A&F University, Xianyang 712100, China
[2] State Key Laboratory of Animal Nutrition and Feeding, Institute of Animal Science, Chinese Academy of Agricultural Sciences, Beijing 100193, China
* Correspondence: yaojunhu2004@sohu.com (J.Y.); zhangjie09@caas.cn (J.Z.)

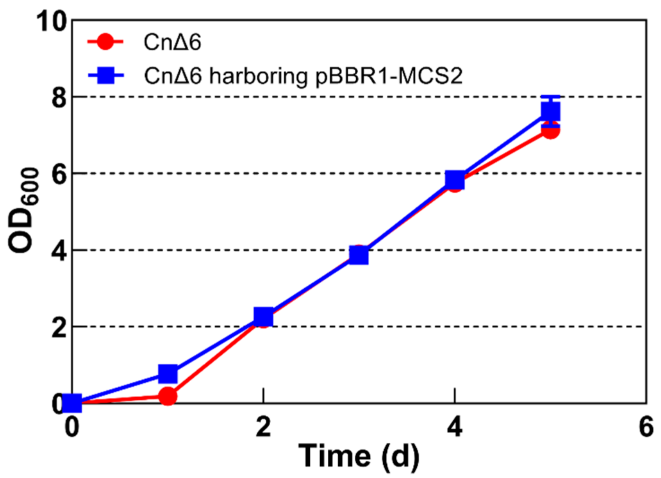

Figure S1. Effect of the empty plasmid pBBR1-MCS2 on the growth of *C. necator* in CnMM with fructose as sole carbon source.

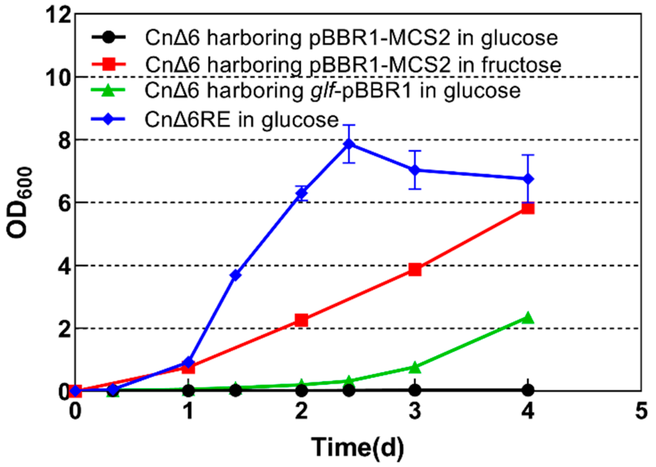

Figure S2. Growth curves of *C. necator* with different strategies to utilize glucose in CnMM. Strain CnΔ6RE, modifying N-acetylglucosamine transport system by mutation of NagE(G265R) and deletion of *nagR* gene, showed better growth compared with strain CnΔ6, which expressing exogenous *glf* gene in plasmid.

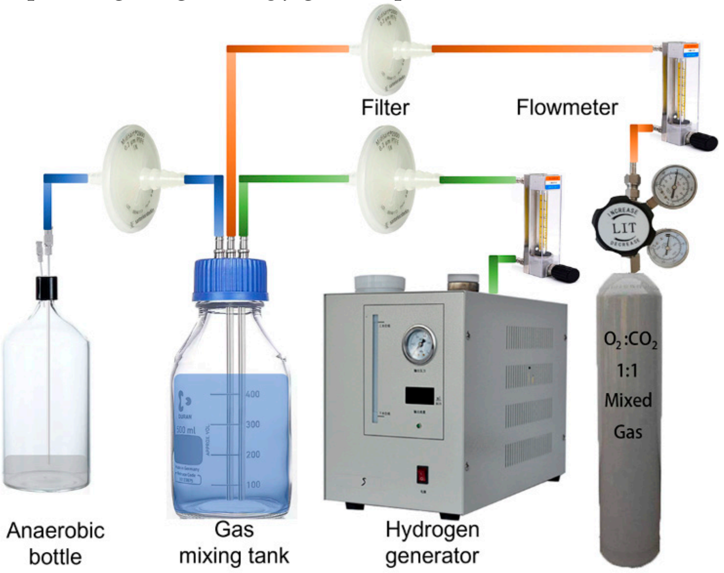

Figure S3. The mixed gas charging system for autotrophic fermentation.

Table S1. Primers for plasmids construction in this study.

Functions	Template	Primer	Sequences
**pK18-*phaC1AB1***			
Upstream homologous arm	*C. necator*	PHB-up-F	aacagctatgacatgattactgatcgaagccacgctgtcacg
		PHB-up-R	tggttgaaccattgcaacgcacgcgctgtcaatg
Downstream homologous arm	*C. necator*	PHB-down-F	tgcgttgcaatggttcaaccagtcggcagcc
		PHB-down-R	aggtcgactctagaggatcctgcatggcatggccgtagaag
**pK18-*nagR***			
Upstream homologous arm	*C. necator*	nagR-up-F	aacagctatgacatgattaccatcatcttcatggtcgcgg
		nagR-up-R	atggatcatatggaattcaagcagtagtgcccggcacg
Downstream homologous arm	*C. necator*	nagR-down-F	actgcttgaattccatatgatccattgcgaatcctcgtag
		nagR-down-R	aggtcgactctagaggatccaggccgtcgccgaacatgcc
**pK18-$nagE_{G793C}$**			
Upstream homologous arm	*C. necator*	nagE-up-F	aacagctatgacatgattacatgggcgtgctggccggcatc
		nagE-up-R	agaacagcatgcggccaaccagcgcgcgc
Downstream homologous arm	*C. necator*	nagE-down-F	tggttggccgcatgctgttctcgatggc
		nagE-down-R	aggtcgactctagaggatccgtacgcccagtgccttgag
**pK18-*alaE-ldhA1A2***			
Upstream homologous arm	*C. necator*	ldhA12-up-F	aacagctatgacatgattacatgacctcaccctcgagttcggtg
		ldhA12-up-R	ttgcaacgcacgcgctgtcaaatcatcgagtagccgaggtcg
P_{phaC1} promoter	*C. necator*	ldhA12-P_{phaC1}-F	ttgacagcgcgtgcgttgcaag
		P_{phaC1}-alaE-R	gatttgattgtctctctgccgtc
alaE gene	*E. coli*	P_{phaC1}-alaE-F	acggcagagagacaatcaaatcatgttctcaccgcagtcacgc
		alaE-ldhA12-R	tccgcgacaattcaggcttttacctgctggtaac
Downstream homologous arm	*C. necator*	ldhA12-down-F	aaagcctgaattgtcgcggaccatgatgaaatggg
		ldhA12-down-R	aggtcgactctagaggatcccaacatgtctgtgtggagtctgc
***gnd*-pBBR1**			
P_{phaC1} promoter	*C. necator*	P_{phaC1}-F	atgaccgcggccgctgtatcttgacagcgcgtgcgttgcaag
		P_{phaC1}-gnd-R	tgcttggacatgatttgattgtctctctgccg
gnd gene	*E. coli*	P_{phaC1}-gnd-F	acaatcaaatcatgtccaagcaacagatcggc
		gnd-R	aggatccccgggtaccgagctcttaatccagccattcggtatgg
***pfkA*-pBBR1**			
P_{phaC1} promoter	*C. necator*	P_{phaC1}-F	atgaccgcggccgctgtatcttgacagcgcgtgcgttgcaag
		P_{phaC1}-pfkA-R	tcttaatcatgatttgattgtctctctgccg
pfkA gene	*E. coli*	P_{phaC1}-pfkA-F	acaatcaaatcatgattaagaaaatcggtgtgttg
		pfkA-R1	aggatccccgggtaccgagctcttaatacagtttttcgcgcag
***gnd-pfkA*-pBBR1**			
P_{phaC1} promoter	*C. necator*	gnd-P_{phaC1}-F	tggattaagagctcggtaccttgacagcgcgtgcgttgcaag
		P_{phaC1}-pfkA-R	tcttaatcatgatttgattgtctctctgccg
pfkA gene	*E. coli*	P_{phaC1}-pfkA-F	acaatcaaatcatgattaagaaaatcggtgtgttg
		pfkA-R2	acgtcgactctagaggatcccttaatacagtttttcgcgcag
**$alaD_{gs}$-pBBR1**			
P_{phaC1} promoter	*C. necator*	P_{phaC1}-F	atgaccgcggccgctgtatcttgacagcgcgtgcgttgcaag
		P_{phaC1}-R	tgatttgattgtctctctgccg

alaD$_{gs}$ gene	Synthetic	P$_{phaC1}$-D$_{gs}$-F	acggcagagagacaatcaaatcatgaagatcggcatcccgaagg
		D$_{gs}$-R	tccccgggtaccgagctcgaattcttagccatgcagcagcgagtg
***alaD*$_{ls}$-pBBR1**			
P$_{phaC1}$ promoter	*C. necator*	P$_{phaC1}$-F	atgaccgcggccgctgtatcttgacagcgcgtgcgttgcaag
		P$_{phaC1}$-R	tgatttgattgtctctctgccg
alaD$_{ls}$ gene	Synthetic	P$_{phaC1}$-D$_{ls}$-F	acggcagagagacaatcaaatcatgaagatcggcatcccgaagg
		D$_{ls}$-R	tccccgggtaccgagctcgaattcttactgaatcagctcatccac
***alaD*$_{NADPH}$-pBBR1**			
P$_{phaC1}$ promoter	*C. necator*	P$_{phaC1}$-F	atgaccgcggccgctgtatcttgacagcgcgtgcgttgcaag
		P$_{phaC1}$-R	tgatttgattgtctctctgccg
alaD$_{NAPDH}$ gene	Synthetic	P$_{phaC1}$-D$_{NADPH}$-F	acggcagagagacaatcaaatcatgatcatcggcgtgccgaag
		D$_{NADPH}$-R	tccccgggtaccgagctcgaattcttaggcgccggccaccgacg
**araP*alaD*$_{gs}$-pBBR1**			
*araC*P$_{BAD}$ promoter	*E. coli*	araCP$_{BAD}$-F	atgaccgcggccgctgtatcttatgacaacttgacggctac
		araCP$_{BAD}$-R	atgtatatctccttcttaaaagatc
alaD$_{gs}$ gene	Synthetic	ara-D$_{gs}$-F	tcttttaagaaggagatatacatatgaagatcggcatcccgaag
		D$_{gs}$-R	tccccgggtaccgagctcgaattcttagccatgcagcagcgagtg
**araP*alaD*$_{ls}$-pBBR1**			
*araC*P$_{BAD}$ promoter	*E. coli*	araCP$_{BAD}$-F	atgaccgcggccgctgtatcttatgacaacttgacggctac
		araCP$_{BAD}$-R	atgtatatctccttcttaaaagatc
alaD$_{ls}$ gene	Synthetic	ara-D$_{ls}$-F	tcttttaagaaggagatatacatatgaagatcggcatcccgaag
		D$_{ls}$-R	tccccgggtaccgagctcgaattcttactgaatcagctcatccac
**araP*alaD*$_{NADPH}$-pBBR1**			
*araC*P$_{BAD}$ promoter	*E. coli*	araCP$_{BAD}$-F	atgaccgcggccgctgtatcttatgacaacttgacggctac
		araCP$_{BAD}$-R	atgtatatctccttcttaaaagatc
alaD$_{NAPDH}$ gene	Synthetic	ara-D$_{NADPH}$-F	ttttaagaaggagatatacatatgatcatcggcgtgccgaag
		D$_{NADPH}$-R	tccccgggtaccgagctcgaattcttaggcgccggccaccgacg
***alaD-gnd*-pBBR1**			
P$_{phaC1}$-*gnd* fragment	*gnd*-pBBR1	Pgnd-F	aattcgagctcggtacccggatgaccgcggccgctgtatcttg
		Pgnd-R	gtgacgtcgactctagaggatccttaatccagccattcggtatg
***alaD-pfkA*-pBBR1**			
P$_{phaC1}$-*pfkA* fragment	*pfkA*-pBBR1	Pgnd-F	aattcgagctcggtacccggatgaccgcggccgctgtatcttg
		PpfkA-R	gtgacgtcgactctagaggatccttaatacagttttttcgcgcagtcc
***alaD-gnd-pfkA*-pBBR1**			
P$_{phaC1}$-*gnd*-P$_{phaC1}$-*pfkA* fragment	*gnd*-pfkA-pBBR1	Pgnd-F	aattcgagctcggtacccggatgaccgcggccgctgtatcttg
		PpfkA-R	gtgacgtcgactctagaggatccttaatacagttttttcgcgcagtcc
